# The Role of Transthoracic Ultrasound in the Study of Interstitial Lung Diseases: High-Resolution Computed Tomography Versus Ultrasound Patterns: Our Preliminary Experience

**DOI:** 10.3390/diagnostics11030439

**Published:** 2021-03-04

**Authors:** Donato Lacedonia, Giulia Scioscia, Angelamaria Giardinelli, Carla Maria Irene Quarato, Ennio Vincenzo Sassani, Maria Pia Foschino Barbaro, Federica Maci, Marco Sperandeo

**Affiliations:** 1 Department of Medical and Surgical Sciences, Institute of Respiratory Diseases, University of Foggia, 71122 Foggia, Italy; donato.lacedonia@unifg.it (D.L.); jengiard87@hotmail.it (A.G.); carlamariairene.quarato@gmail.com (C.M.I.Q.); mariapia.foschino@unifg.it (M.P.F.B.); fede.maci91@gmail.com (F.M.); 2 Institute of Respiratory Diseases, Policlinico “Riuniti” di Foggia, 71122 Foggia, Italy; 3 Department of Radiology, Policlinico “Riuniti” di Foggia, 71122 Foggia, Italy; ennio.sassani@tiscali.it; 4 Unit of Interventional and Diagnostic Ultrasound, Department of Internal Medicine, IRCCS Fondazione “Casa Sollievo della Sofferenza”, 71013 San Giovanni Rotondo, Italy; sperandeomar@gmail.com

**Keywords:** transthoracic ultrasound, high-resolution computed tomography, interstitial lung diseases, hyperechoic pleural line, screening tool

## Abstract

Transthoracic ultrasound (TUS) is a readily available imaging tool that can provide a quick real-time evaluation. The aim of this preliminary study was to establish a complementary role for this imaging method in the approach of interstitial lung diseases (ILDs). TUS examination was performed in 43 consecutive patients with pulmonary fibrosis and TUS findings were compared with the corresponding high-resolution computed tomography (HRCT) scans. All patients showed a thickened hyperechoic pleural line, despite no difference between dominant HRCT patterns (ground glass, honeycombing, mixed pattern) being recorded (*p* > 0.05). However, pleural lines’ thickening showed a significant difference between different HRCT degree of fibrosis (*p* < 0.001) and a negative correlation with functional parameters. The presence of >3 B-lines and subpleural nodules was also assessed in a large number of patients, although they did not demonstrate any particular association with a specific HRCT finding or fibrotic degree. Results allow us to suggest a complementary role for TUS in facilitating an early diagnosis of ILD or helping to detect a possible disease progression or eventual complications during routine clinical practice (with pleural line measurements and subpleural nodules), although HRCT remains the gold standard in the definition of ILD pattern, disease extent and follow-up.

## 1. Introduction

Interstitial lung diseases (ILDs) are a heterogeneous group of lung diseases characterized by an abnormal and progressive scarring reaction of the interstitium, resulting in impaired gas exchange and in a restrictive (spirometric) alteration. Their etiology can be of a primitive nature or secondary (e.g., connective diseases, hypersensitivity and drug toxicity).

Clinically, an early diagnosis and therapeutic management is very relevant because, depending on the specific case, the removal of the triggering cause, the use of corticosteroids and the availability of anti-fibrotic treatments may slow disease progression and improve the prognosis [1,2].

Current international guidelines recommend that, for each patient suspected of having an ILD, a multidisciplinary conference in a dedicated Lung Unit should take place to discuss clinical, functional and high-resolution computed tomography (HRCT) findings for diagnostic and therapeutic decision-making [3,4]. High-resolution computed tomography (HRCT) represents the gold standard in the diagnosis of ILDs, allowing the detection of any interstitial fibrotic transformation at a relatively early stage, with some radiological patterns of presentation being very typical. Lung function tests help in monitoring the progression and in determining the severity of disease.

In the last 20 years, the feasibility of transthoracic ultrasound (TUS) has opened up large areas of application and interest in pleuropulmonary US, involving virtually all branches of medicine. Nowadays, almost each medical specialty has its own lung area [5].

Although there is not a standardized role in international guidelines for TUS examination in ILDs, this imaging technology is advantageous in terms of non-invasiveness and safety, is suitable for a quick real-time evaluation and is readily available for all clinicians in all hospital wards.

As ultrasound propagation speed in the aerated lungs is of only 440 m/s, more than 96% of the ultrasound beam (with an initial speed of 1540 m/s) is reflected at the chest wall/lung air interface and by the skeletal structures of the thoracic cage, allowing only the 70% of the pleural surface to be explored [6,7,8]. Despite these limitations inherent in the method, any process involving the subpleural pulmonary interstice (i.e., fibrotic thickening or nodules) may be detected by TUS if it is adherent to the accessible pleural surface [7,8,9].

Several studies have already investigated the role of ultrasound in ILDs [10,11,12,13,14]. Reported findings of pulmonary fibrosis include: (1) a regular or irregular thickening (>3.0 mm) of the hyperechoic pleural line; (2) an irregular and/or fragmented and/or blurred aspect of the hyperechoic pleural line; (3) an increase in the number (>3) of vertical artifacts (i.e., the so called “B-lines”) between two ribs in a single scan; (4) evidence of subpleural nodulations [9,10,12,13,15].

In particular, in ILD associated with connective diseases, such as systemic sclerosis, in which the fibrotic process generally originates in the subpleural basal-posterior lung interstitium (which is easily accessible to ultrasound), TUS has been suggested as a valid screening complementary tool [15]. Indeed, in these patients, TUS has the potential to indicate both early pulmonary involvement and eventual ILD progression during the time course of the disease, acting as a timely indicator for the execution of a control chest computed tomography during routine follow-up [10,13,14]. For such purposes, the measurement of the thickness of the hyperechoic pleural line seems to be the most rewarding method for a reproducible TUS assessment, showing good correlation with the degree of fibrosis [10].

In this context, the aim of this preliminary study was to establish a possible role for TUS in the diagnostic approach of ILDs. With this purpose, we tried to assess a correspondence between HRCT patterns and ultrasound findings in patients suffering from different ILDs with several degrees of lung involvement (minimal, mild, moderate and severe). Additionally, we tried to recognize a possible correlation between the thickness of the hyperechoic pleural line and functional parameters, such as forced vital capacity (FVC), diffusing lung capacity for carbon monoxide (DLCO), meters traveled and Nadir SpO_2_ during the 6-minutes walking test (6mWT), whose impairment is associated with the severity of the lung disease.

## 2. Materials and Methods

In this single-center observational study, we enrolled 43 consecutive patients diagnosed with ILD (32 M and 11 F, mean age: 70.77 ± 8.32 years), who were followed-up in our ILD outpatient clinic.

Diagnoses were made, according to international guidelines, on the basis of clinical presentation, HRCT findings, pulmonary function tests (PFTs), fiberbronchoscopic findings and bronchoalveolar lavage and after reaching consensus in a multidisciplinary Lung Unit conference [3,4].

This study was carried out according to the principles of the Declaration of Helsinki and was approved by the local ethics committee (institutional review board approval N 17/CE/June 12, 2014). All recruited patients gave their written informed consent.

### 2.1. High-Resolution Computed Tomography (HRCT)

Each patient underwent a control chest HRCT approximately one week prior to the scheduled visit. HRCT examinations were performed using a multi-detector CT scanner with 64 channels (Toshiba, Tokyo, Japan). The detailed parameters for CT acquisition were as follows: tube voltage, 120 kVp; tube current, standard (reference mAs, 60–120); slice thickness, 0.5 mm; reconstruction interval, 0.5–1.0 mm. All CT images were acquired at full inspiration, with the patient in the supine position and without contrast medium.

HRCT images were carefully examined for the following ILD findings: ground-glass opacity (GG) = an area of increased parenchymal attenuation with preserved bronchial and vascular markings; reticular abnormalities = a fine network of linear opacities within lobules; nodularity = nodular opacities with a maximum diameter of 3 cm in the peribroncho-vascular interstitial space, in the interlobular septa and in the subpleural interstitial space; traction bronchiectasis = dilatation of bronchial tree with peribronchial wall thickening; honeycombing (HC) = clustered air-filled cyst with dense walls. The dominant pattern for each patient was classified into four categories: ground glass, reticular, nodular, honeycombing [16]. In most cases, more than one HRCT pattern was recognized in the same patient.

Each lung was divided into the following three zones: upper (lung apex to aortic arch); middle (aortic arch to inferior pulmonary veins); and lower (inferior pulmonary veins to lung bases) [17]. Each zone of right and left lung was assessed for the degree of involvement and semi-quantitatively scored as absent (0%), minimal (1–25%), mild (26–50%), moderate (51–75%) and severe (>76%). Patients were classified as having a minimal, mild, moderate and severe degree of fibrosis according to the higher zonal score recorded.

Predominantly basal and subpleural reticular abnormalities associated with peripheral traction bronchiectasis/bronchiolectasis with and without the presence of honeycombing were classified as a “definitive” or “probable” Usual Interstitial Pneumonia (UIP) pattern, respectively [4].

Extensive bilateral patchy GG admixed with reticulation and traction bronchiectasis/bronchiolectasis in a peripheral, subpleural and basal lung localization with subpleural sparing were recognized as a Non-Specific Interstitial Pneumonia (NSIP) pattern [3].

The presence of centrilobular and/or paraseptal emphysema in the upper lobes and pulmonary fibrosis in the lower lobes was classified as “combined pulmonary fibrosis and emphysema” (CPFE) syndrome [3,18].

Coexisting lung fibrosis and signs of bronchiolar obstruction (i.e., ill-defined centrilobular nodules and mosaic attenuation) allowed for a diagnosis of fibrotic Hypersensitivity Pneumonitis (HP) [19].

Other types of radiological interstitial abnormalities that did not fall under the international recommendations were regarded as undefined lung fibrosis [3].

### 2.2. Transthoracic Ultrasound (TUS)

TUS examination was performed by an ultrasound scanner, MyLab Five (Esaote, Genova, Italy), equipped with a low-frequency convex probe (3.5–5 MHz) and a high-definition linear transducer (8–12.5 MHz), using the correct setting for the adult thoracic study (gain: max 50%, focus pointed at the hyperechoic pleural line, activation of the tissue harmonic imaging).

Patients’ chests were examined with intercostal longitudinal and transversal scans from the lung base to the apex, posteriorly (along the para-vertebral, hemi-scapular and posterior-axillary lines), laterally (along the middle-axillary line) and anteriorly (along anterior-axillary, hemi-clavicular and para-sternal lines) in a sitting position.

Ultrasound scans were focused on the following assessments: thickness of the hyperechoic pleural line, qualitative ultrasound features of the hyperechoic pleural line (irregular and/or fragmented and/or blurred), presence of vertical artifacts (>3 or ≤B-lines) and eventual subpleural nodules (number, location, shape and size).

Previous evaluations on healthy subjects have shown that upper limits of normal for pleural line thickness with a low-frequency convex probe (3.5–5 MHz) are 1.4–2.8 mm, while, when using a high-definition linear transducer (8–12.5 MHz), they are 0.6–1.8 mm [10]. Therefore, in the present study, all the measurements of the hyperechoic pleural line’s thickness for subsequent data analysis were provided exclusively using convex probes. A conventional cut-off of 3.0 mm was used for defining normal (≤3.0 mm) or increased (>3.0 mm) thickness of the pleural line.

To complete the ultrasound examination, a high-frequency linear probe (8–12.5 MHz) was used in order to obtain a more detailed definition of the qualitative echographic characteristics of the hyperechoic pleural line. Hyperechoic pleural line abnormalities were noted if, in contrast to a normal thin, smooth aspect, it appeared irregularly thickened (irregularity), showed focal interruptions (fragmented) or presented less definite contour (blurred) [9,20].

B-lines, or vertical artifacts, were defined as continuous and parallel hyperechoic stripes, arising from the pleural line and extending indefinitely along the direction of the US beam on the screen [7]. As B-lines are a dynamic LUS artifact, moving and potentially changing in number and appearance over the respiratory cycle, their exact count and description was not considered in this study, because this type of approach lacks reproducibility and scientific objectivity [21,22]. Therefore, an increased number of such artifacts was semi-quantitatively assessed by the presence of more than 3 B-lines [13].

Subpleural nodules were defined as subpleural hypo-echoeic small lesions, round or oval in shape, interrupting the hyperechoic pleural line [9].

TUS examinations were performed and interpreted by 2 sonographers with at least 5 years of experience. The HRCT pattern and the degree of fibrosis of each patient were blinded to the sonographer during the exam. Inter-observer variability was assessed by an expert sonographer with 30 years of experience, 1-day apart, taking repeated measures on the recorded videoclips for each subject. Coefficients of variation (CV%) showed that the differences between measurements were small (0.6–5.8%) and Cohen’s kappa indicated a good agreement (0.60–0.80).

### 2.3. Pulmonary Function Tests (PFTs)

During the planned follow-up visit, each patient underwent pulmonary function tests using a spirometer (Sensormedics, Yorba Linda, CA, USA). Forced expiratory volume in 1 s (FEV_1_) and forced vital capacity (FVC) were measured with standard spirometry. The best value of three maneuvers was expressed as a percentage of the predicted normal value [23].

The diffusing capacity of the lung for carbon monoxide (DLCO) was measured with the “single breath” technique and corrected for hemoglobin and carbon monoxide (CO) levels. The results were registered as percentages of predicted values [24].

### 2.4. Six-Minute Walking Test (6mWT)

At the end of pulmonary function tests, each patients performed the 6-minutes walking test (6mWt) and the following variables were obtained: the distance walked during 6 min of time (meters traveled) and the nadir value of the peripheral oxygen saturation (Nadir SpO_2_%) reached during the test [25].

### 2.5. Statistical Analysis

Data are presented as means ± standard deviation (SD) for continuous variables and as number (*n*) and percentage (%) for descriptive variables.

Pleural line thickness measurements, taken over the entire chest surface, were recorded. Statistical analysis was performed using measures taken in the area of the chest wall corresponding to the most highly involved lung zone of each patient (i.e., at the posterior-basal level in almost all cases). The same was done in assessing a finding of >3 B-lines.

Unpaired Student’s *t*-test was used for comparisons of pleural line thickness measurements between patients with and without honeycombing.

One-way ANOVA test was used to assess the difference between different HRCT patterns (ground glass, honeycombing, mixed pattern) and between different degrees of lung involvement. In addition, a pairwise post-hoc Tukey test was performed to determine whether there was a difference between the mean or the frequency of all possible pairs.

Pearson correlation coefficients (*r*) were used to assess the association between thickness of the pleural line and functional parameters (FVC%, DLCO%, meters traveled and Nadir SpO_2_ during 6mWT).

Significance was established at a *p*-value < 0.05.

## 3. Results

Demographic, clinical, functional and imaging data of the 43 study patients are summarized in Table 1.

In total, 28 patients had an HRCT UIP pattern and received a final diagnosis of Idiopathic Pulmonary Fibrosis (IPF), four patients presented a CPFE syndrome, two patients had an HRCT NSIP pattern, four patients received a diagnosis of fibrotic HP and five patients had an undefined ILD.

According to the higher zonal score recorded on HRCT, four patients were classified as having a minimal degree of fibrosis, 16 as mild, 10 as moderate and 13 as severe.

The higher degree of lung fibrosis was recorded in the lower lung zones in almost all the patients (93%). Only three patients (7%) showed a higher involvement in the middle zones. Such patients had a diagnosis of fibrotic HP.

In all of our patients, TUS was able to assess a thickening of the hyperechoic pleural line in correspondence with the areas affected by subpleural fibrosis at HRCT. The average thickening of the pleural line measured with a low-frequency convex probe on the chest wall in the area corresponding to the most highly involved lung zone of each patients was 4.69 mm.

Table 2 shows the average thickening of the pleural line (measured with convex probe) in the different groups of patients compared to each other.

**Figure 1:**
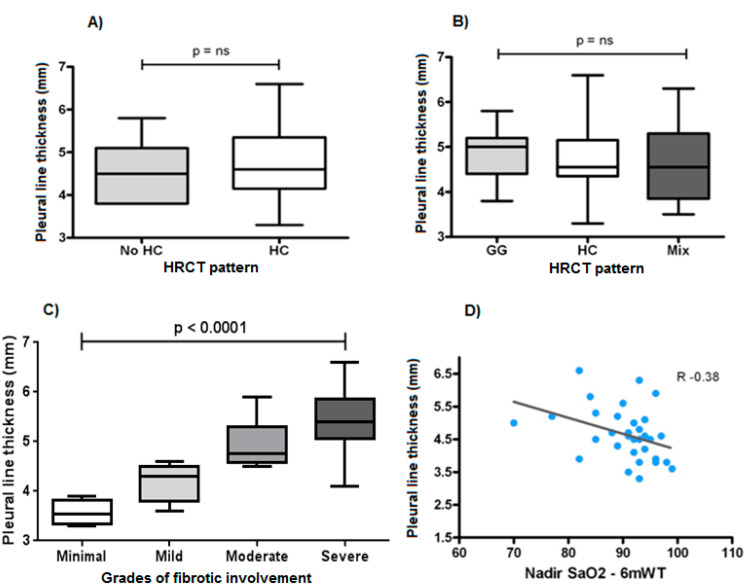
(**A**) Thickness of the hyperechoic pleural line between honeycombing (HC) and no honeycombing (no HC) HRCT pattern (*p* > 0.05). (**B**) Thickness of the hyperechoic pleural line between different HRCT patterns (GG: ground glass, HB: honeycombing, Mix: mixed patterns) (*p* > 0.05). (**C**) Thickness of the hyperechoic pleural line between patients with different grades of fibrotic involvement (*p* < 0.0001). (**D**) Correlation between the thickness of the hyperechoic pleural line and the Nadir value of SaO_2_% reached during 6-min walking test (6mWT) (*r* −0.38).

According to HRCT findings, 32 patients had subpleural honeycombing and 11 patients showed subpleural fibrotic involvement without honeycombing. No difference in terms of thickness of the hyperechoic pleural line between patients with and without honeycombing was recorded (*p* > 0.05) (Figure 1A).

More specifically, five patients showed a predominant pattern of subpleural ground glass, 28 patients had a predominant pattern of subpleural honeycombing and 10 patients showed a mixed pattern where no clear prevalence of a determinate HRCT finding was noticed (ground glass, reticular opacities, nodularity or honeycombing).

The amount of thickening of the hyperechoic pleural line was found to be independent of the dominant HRCT pattern (ground glass, honeycombing, mixed pattern) and proved to be superimposable in the three groups (*p* > 0.05) (Figure 1B).

Nevertheless, the thickness of the hyperechoic pleural line showed a statistically significant increase between different degrees of lung involvement at HRCT (*p* < 0.001) (Figure 1C).

Relating the thickness of the hyperechoic pleural line measured in the thoracic area corresponding to the higher involved lung zone at HRCT with functional parameters, a negative correlation emerged with the forced vital capacity, FVC% (*r* = 0.21), the diffusion lung capacity of carbon monoxide, DLCO% (*r* = 0.24) and the meters traveled during 6mWT (*r* = 0.31); the strongest statistically significant relationship was that between the thickness of the hyperechoic pleural line and the Nadir value of SaO_2_% reached during 6mWt (*r* = 0.38) (Figure 1C).

**Figure 2:**
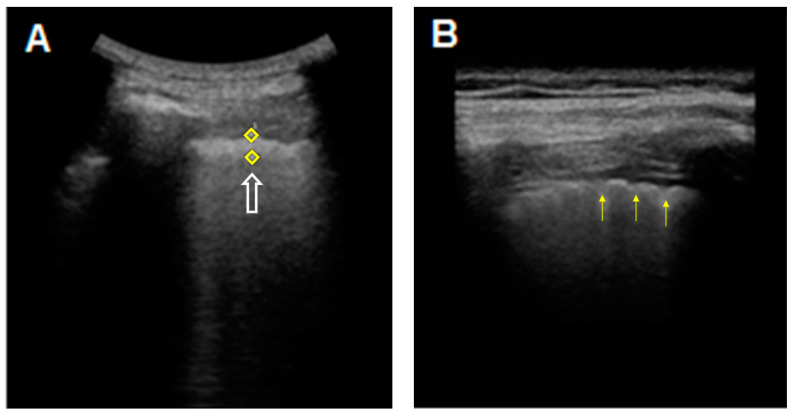
TUS images of the hyperechoic pleural line in a patient with an HRCT pattern of Usual Interstitial Pneumonia (UIP). (**A**) The hyperechoic pleural line (white arrow) appears thickened (5.1 mm) when measured with a middle/low-frequency convex probe (3.5–5 MHz). (**B**) The thickened (2.4 mm), irregular, fragmented and blurred appearance (yellow arrows) becomes more evident when using a high-frequency linear probe (8–12.5 Mhz).

On TUS examination with a high-frequency linear probe (8–12.5 MHz), in our patients with ILDs (with only one exception), the hyperechoic pleural line appeared not only thickened (>2 mm), but also fragmented and blurred; moreover the irregularities of the pleural line appeared more pronounced in the areas of greater fibrotic involvement (Figure 2).

**Figure 3:**
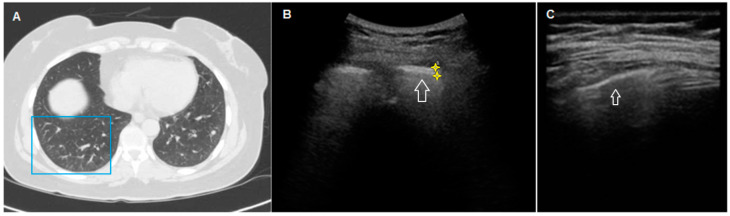
HRCT and TUS scan of a 54-year-old woman with familiarity for pulmonary fibrosis. In (**A**), HRCT scan shows a diffuse bilateral peripheral increase in pulmonary density (ground glass opacity) with widespread parenchymal nodular lesions (maximum size: 7 mm), in greater numbers in the lower lobes. In (**B**), TUS scan with a low-frequency convex probe (3.5–5 MHz) (in the chest area corresponding to the blue box in (**A**) shows a thickened pleural line (white arrow)). In (**C**), TUS scan with a high-frequency linear probe (8–12.5 MHz) at the same level does not show a clearly blurred and fragmented aspect.

In only one patient, an extensive pure ground glass HRCT pattern seemed to not determine the clear irregularity and fragmentation of the hyperechoic pleural line, which, conversely, appeared equally thickened but more linear and less irregular and blurred (Figure 3). Unfortunately, this last finding was not supported by a sufficiently large number of observed cases.

The presence of >3 B-lines between two ribs in a single scan was verified in 38 patients (86%). However, this finding was not able to discriminate between different degrees of subpleural interstitial involvement (*p* > 0.05) (Table 3).

Five patients showed a reticulo-nodular pattern at HRCT. In four patients, this pattern was associated with honeycombing. TUS detected subpleural nodules in 32 patients.

Among these 32 patients, three presented a reticulo-nodular pattern with honeycombing, 23 presented a reticular pattern with honeycombing and five presented a reticular pattern without honeycombing at HRCT (Table 4).

## 4. Discussion

The main objective of our preliminary study was to evaluate whether the use of TUS can represent a useful complementary tool for a first evaluation of patients with suspected or known ILD during routine clinical visits.

Our experience seems to suggest that the signs of pulmonary fibrosis shown on chest HRCT correlate with the following findings on TUS: (1) increased thickness of the pleural line; (2) an irregular, fragmented and/or blurred appearance of the pleural line; (3) >3 B-lines and (4) subpleural nodules. However, the significance of these TUS findings must be thoroughly discussed and carefully assessed by integrating the patient’s clinical picture, in order to avoid hasty conclusions and misdiagnoses.

The hyperechoic “pleural line” is an ultrasound imaging error (or “artifact”) resulting from the large difference in acoustic impedance between chest wall soft tissues and pulmonary air content [7,8]. Considering that the actual thickness of pleural membranes under the microscope is only around 150–200 μm, it has to be regarded as a virtual image that has no direct anatomical equivalent [26]. Due to the physical nature of the artifact, the measurement of its thickness may slightly differ under different frequencies (i.e., type of probe) and settings of the ultrasound scanner used [7]. For this reason, as mentioned in the Materials and Methods section, all measures considered for analysis in this study were taken using a convex probe, while a high-frequency linear probe was used for qualitative study (i.e., in assessing its aspect).

In a previous evaluation on 200 healthy subjects (i.e., with no acute or chronic respiratory diseases, or rheumatic disease), our group recorded a mean pleural line thickness of 1.4–1.1 mm (range 1.4–2.8 mm) using a low-frequency convex probe (3.5–5 MHz) [10]. In the present case series, we detected an increased thickness of the hyperechoic pleural line (>3 mm) in all the chest areas corresponding to zones of lung fibrotic involvement. However, the thickness of the pleural line was not able to detect honeycombing areas or to discriminate between different HRCT patterns.

On the one hand, this evidence highlights how TUS assessment of the pleural line is non-specific and HRCT remains the gold standard in the proper study of an ILD. Indeed, while HRCT can be virtually used to explore all of the lung, TUS can at best explore no more than 70% of the most superficial lung pleura, with the risk of missing or underestimating disease extent [7]. Furthermore, it should be remembered that some false-positive conditions, such as subpleural bronchiectasis, cysts, blebs and emphysema, may generate an irregular thickening of the hyperechoic pleural line and simulate a fibrotic interstitial lung disease [9].

On the other hand, however, in the clinical context of respiratory symptoms and pulmonary function test impairment, TUS seems to be able to reasonably suggest the presence of disease. In such cases, the finding of an increased pleural line thickness at TUS may help to define a timely HRCT assessment in order to reach an early diagnosis.

In addition, in our experience, the measurement in mm of the hyperechoic pleural line showed a statistically significant increase between different degrees of lung involvement at HRCT. This may suggest a TUS role as an easily accessible tool for monitoring the evolution of the disease during routine follow-up (i.e., in assessing an increase in thickness of the hyperechoic pleural line compared to the previous control). Obviously, also in this scenario, the pivotal role of HRCT in ascertaining the actual degree of fibrosis progression and its necessity to confirm a disease progression must not be forgotten.

In all our patients with ILDs (with only one exception), the pleural line appeared not only thickened, but also fragmented and blurred. In particular, pleural line irregularities appeared more pronounced in the areas of greater fibrotic alteration. The qualitative assessment of the hyperechoic pleural line with a high-frequency linear probe may, therefore, strengthen the clinical suspicious.

Respiratory function data were also recorded in our patients. Main causes of DLCO impairment in ILDs are the thickening of the alveolar epithelial–endothelial barrier and the mismatching of ventilation and perfusion. A reduced DLCO% is the most sensitive parameter for detecting early ILD before lung volumes become decreased and for monitoring response to therapy and disease progression [27]. However, the DLCO test is characterized by an intrinsically reduced reproducibility that may bias its reliability during follow-up [28]. FVC% is certainly the simplest and least variable parameter to employ in the follow-up of ILDs [29]. Nevertheless, to stage the disease and evaluate prognosis, in addition to any static determination, it is equally important to quantify the functional reserve of the lungs. The distance covered during a sub-maximal exercise test, such as 6-min walking test, allows the evaluation of exercise tolerance. Furthermore, desaturation during the test is an important negative prognostic index, also giving indication to oxygen therapy under stress [30]. Interestingly, relating the thickness of the hyperechoic pleural line with functional parameters (i.e., FVC%, DLCO%, meters traveled and Nadir value of SaO_2_% during 6mWT), we recorded a negative, albeit very weak, correlation. This result might support, also from a functional point of view, the notion that an increased thickness of the pleural line reflects a greater fibrotic involvement of the peripheral (subpleural) pulmonary interstice.

The finding of more than three B-lines between two ribs in a single scan was verified in almost all of our patients. Its presence was highly prevalent at all severity grades of pulmonary fibrosis but was not able to discriminate between different degrees of pulmonary involvement, thus demonstrating a role of generic sign of the presence of disease. However, this finding must be judged very carefully. The presence of more than three B-lines alone has never truly gained widespread scientific acceptance as a marker of “interstitial fibrosis” [21,31]. Indeed, in the literature, an increased number of B-lines has been described in several pathological conditions, ranging from lung fluid accumulation (i.e., heart failure [32] or end-stage renal disease accompanied by pulmonary congestion [33]), lung injury and/or inflammation (i.e., pulmonary contusion [34], acute respiratory distress syndrome [35], pneumonia [36], acute exacerbation of chronic obstructive pulmonary disease (COPD) [37], acute bronchial asthma [38,39], neoplastic lymphangitis [40]), to pulmonary fibrosis [9,10,12]. Moreover, no statistical significance has been attributed to B-line number in distinguishing between the ‘‘wet’’ lungs of patients affected by acute pulmonary edema and other primary pulmonary conditions [41].

The reason that B-lines increase in all these pathological conditions in which the proportion between air, liquid film and interstitial tissue is changed may be found in a physical phenomenon. In normal conditions, the lung acts as a strong reflector: more than 95% of the US beam is reflected at the chest wall/lung interface, as there is a great difference in acoustic impedance between chest wall soft tissues and pulmonary air at this point. This interface generates the so-called “hyperechoic pleural line” and a series of parallel, equally spaced returning echoes of decreasing intensity, namely “A-line” artifacts. When near the subpleural space, the incident US beam encounters areas of excessive quantity of liquid film or interstitial deposition of collagen tissue, and the impedance gradient between these structures and the surrounding pulmonary air volume creates a phenomenon of resonance. A new ultrasound wave, which is reflected back and towards the transducer, is so produced, generating a narrow, vertically extending artifact of “B-lines” [42,43,44]. On the contrary, if the probe is directly in contact with the lung, thus excluding the differences in acoustic impedance of chest wall soft tissues, the generation of B-lines artifacts does not occur, even in pulmonary areas affected by pulmonary fibrosis. This evidence clearly emerged in a preliminary experience of our group with intraoperative lung ultrasound during video-assisted thoracic surgery (VATS-ILU) [45,46].

Considering that B-lines in TUS are also found in normal subjects (i.e., generally at the bases, where the hydrostatic pressure gives a more fluid-rich interstitium [47]) and that the finding of more than three B-lines is present also in other pathologic conditions, the increased presence of this artifact cannot be regarded as an indicator of lung fibrosis when observed alone. Thus, in case of clinical suspicion of ILD, we have to focus not only on B-lines but also on the pleural line and on other eventually associated pleuropulmonary alterations (e.g., subpleural nodules) during TUS examination. At the same time, an increase in B-lines cannot be safely recommended as a useful sign to confirm disease worsening, because it may underlie also other supervening or transitory conditions (e.g., pulmonary edema, pneumonia, exacerbation of underling respiratory diseases).

Reference studies have used various types of scanners and probes for the counting or visual characterization of such artifacts [11,13,14,48]. However, different frequencies and machine factors may influence the viewable number of these artifacts either in the normal lung or in disease. In particular, high-frequency linear probes (8–12.5 MHz) reduce the number of artifacts; low-frequency convex probes (3–8 MHz) increase the number of artifacts; sectorial cardiac probes (2–3.5 MHz) create a folding in the US beam that generates a higher number of artifacts. Similarly, an excessive total gain and the lack of tissue harmonic imaging are generally associated with the detection of a higher number of B-lines. This implies that clinicians using different scanners and different probes will generally reach different conclusions, if their findings are simply based on the counting or visual characterization of B-lines. Furthermore, as reported elsewhere [7,21,22], the simple change in positioning of the probe with respect to the curvature of the patient’s chest and the patient’s respiratory rate may increase the perceived occurrence of such artifacts, making the evaluation process of B-line number to be at best a subjective “semiquantitative overview” rather than an actual ‘‘measurement’’. These limitations explain why the proposal of B-line counting is contrary to efforts to improve the reliability and objectivity of imaging.

**Figure 4:**
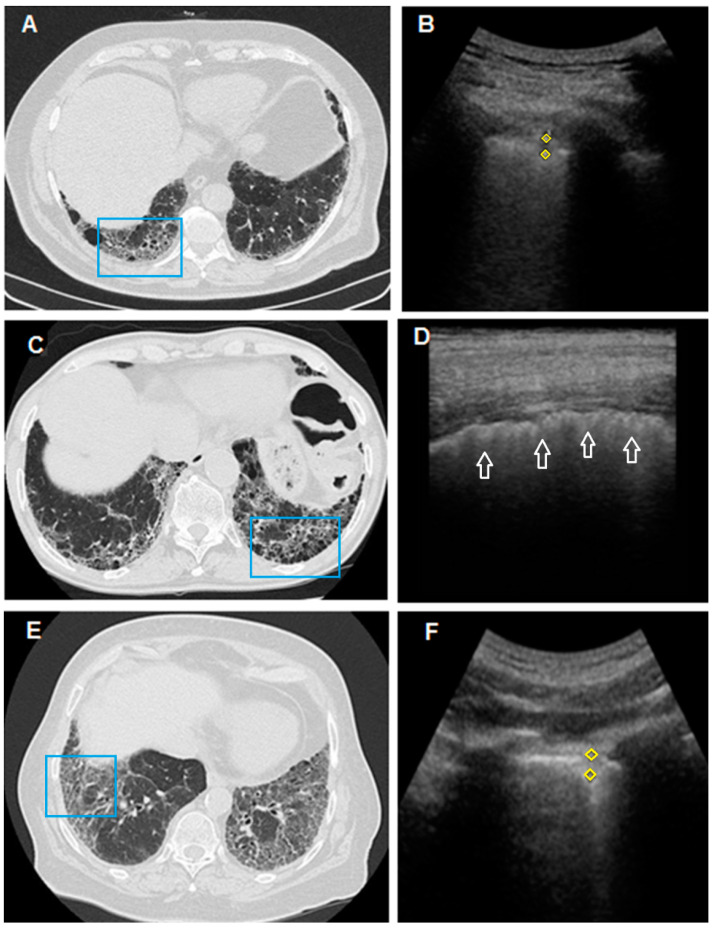
In (**A**), HRCT scan shows bilateral honeycombing, more severe in the right postero-basal area (blue box); In (**B**), TUS scan with convex probe (3.5–5 MHz), in the chest area corresponding to the blue box in (**A**), shows a subpleural hypoechoic area (yellow rhombus) of 4.5 mm (probably a traction cyst). In (**C**), HRCT scan passing through the postero-basal segments shows honeycombing pattern, larger on the left (blue box). In (**D**), TUS scan performed with a linear probe (8–12.5 MHz) in the chest area corresponding to the blue box in (**C**) shows a thickened (>2 mm), irregular, fragmented and blurred hyperechoic pleural line (white arrow), but subpleural nodules are not appreciated. In (**E**), HRCT scan of the thorax passing through the postero-basal segments shows a typical fibrotic distortion of the pulmonary parenchyma, with ground-glass opacity superimposed on a predominant reticular pattern with subpleural and basal distribution and with associated traction bronchiectasis in the absence of a frank honeycombing (UIP “probable”). (**F**) TUS examination performed with a convex probe (3.5–5 MHz) probe in the chest area corresponding to the blue box in (**E**) shows a subpleural hypoechoic nodule of 5.9 mm.

Subpleural nodules represent another frequent finding in patients with fibrotic distortion of the lung parenchyma, especially in the moderate and severe phases of the disease [12,15]. In our study, there was no unequivocal correspondence between subpleural nodules found by TUS and the presence of a nodular pattern or of a more severe honeycombing pattern on HRCT scan. Probably, this ultrasound finding can also be associated with the presence of areas of fibrotic distortion, traction bronchiectasis and bronchiolectasis (Figure 4). Subpleural nodules should, therefore, be regarded as another unspecific TUS finding of lung disease. In this regard, we should add that pulmonary fibrosis is also an independent risk factor for lung cancer. This evidence seems to be particularly true for idiopathic pulmonary fibrosis, where neoplasm has been shown to mainly develop in areas of major fibrosis and peripheral regions [49,50,51]. In this situation, therefore, routine TUS examination might contribute to the identification also of smaller peripheral, suspicious, malignant nodules and the early determination of their nature in further HRCT follow-ups.

The main limitation of our preliminary study is represented by the small number of patients enrolled. However, this limit is in line with the fact that this was the representation of a real-life experience in a dedicated single-center clinic, which makes it more difficult to collect a large number of patients. Nevertheless, this setting is exactly the most suitable for this type of approach, given the availability, non-invasiveness and low cost of TUS examination for an outpatient’s “first look”. Our preliminary results allow us to speculate that, in this context, TUS could facilitate an early diagnosis of ILD or help to detect a possible progression or complication (e.g., suspicious subpleural nodules), suggesting the early execution of more accurate imaging methods (i.e., HRCT). This may help to avoid delays in treatment, thus improving the prognosis of patients with ILD. Furthermore, use of such tool could allow HRCT to be more confidently delayed in cases in which respiratory symptoms and respiratory function tests do not become worse and TUS imaging is stable, thus saving some patients, such as pregnant women, from exposure to unnecessary doses of radiation. Obviously, more large-scale and potentially reproducible studies, including repeated TUS assessments during follow-up, are needed prior to confirming our suggestion.

Clearly, we must emphasize that this type of approach is suitable only for ILDs in which the pulmonary lesions (i.e., honeycombing, reticular opacities, traction bronchiectasis) are typically distributed in peripheral subpleural zones and may be more easily visible on LUS. In other ILDs (e.g., Respiratory Bronchiolitis-Associated Interstitial Lung Disease, Lymphoid Interstitial Pneumonia and Sarcoidosis), the utility of LUS may be less significant because characteristic lesions are mainly distributed along the peribronchiolar, peribronchiovascular and perilymphatic regions.

## 5. Conclusions

In conclusion, results of our preliminary study showed that HRCT patterns produce similar, more or less recognizable, ultrasound artifacts (i.e., pleural line abnormalities, >3 B-lines, subpleural nodules), suggesting, in an appropriate clinical context, the presence of an ILD characterized by a peripheral distribution. The most reliable finding seems to be an increased thickness of the pleural line, showing a concordance with the degree of the disease at HRCT. Therefore, TUS can be suggested to play the role of a useful complementary, non-invasive and easily available “first look” tool in the assessment of early peripheral interstitium involvement or disease progression during outpatients’ clinical follow-up. In fact, in serial checks of patients with a diagnosis of lung fibrosis, in addition to functional data (clinical examination, spirometry and walking test), an increase in the thickness of the hyperechoic pleural line compared to the previous control could lead to the prediction of eventual worsening. In this case, the execution time of an HRCT examination could be anticipated, allowing for early confirmation. Contrastingly, a stable TUS examination may allow a more confident delay of radiological assessment in particular contexts (e.g., pregnancy). Furthermore, TUS may provide useful information about the presence of possible comorbidities, such as pleural effusion, pneumonia and also early tumors adherent to the pleura (i.e., suspicious subpleural nodules). Nevertheless, TUS findings of ILD are highly non-specific and this imaging method has to be regarded only as a complementary tool. HRCT remains the gold standard both in the initial definition of ILD pattern and in the assessment of the disease’s extent during follow-up.

## Figures and Tables

**Table 1:** Characteristics of the 43 patients in the study.

Characteristics	Data
Age, y, mean ± SD	70.77 ± 8.32
*Gender, n (%)*	
Male	32 (74%)
Female	11 (26%)
*Diagnosis, n (%)*	
UIP/IPF	28 (65%)
CPFE	4 (9%)
NSIP	2 (5%)
HP	4 (9%)
Indeterminate ILD	5 (12%)
*Pulmonary function tests, mean ± SD*	
FVC%	83 ± 19
DLCO%	55 ± 14
Meters traveled during 6mWT	383 ± 80
Nadir SaO_2_ 6mWT	91 ± 5
*HRCT patterns, n (%)*	
Honeycombing	32 (75%)
No Honeycombing	11 (25%)
Predominant Ground Glass	5 (12%)
Predominant Honeycombing	28 (65%)
Mixed	10 (23%)
*Degree of fibrosis, n (%)*	
Minimal	4 (10%)
Mild	16 (37%)
Moderate	10 (23%)
Severe	13 (30%)
*Ultrasound findings, n (%)*	
Thickness of the pleural line (>3 mm)	43 (100%)
Irregular/fragmented/blurred pleural line	42 (98%)
>3 B-lines	38 (86%)
Subpleural nodes	32 (74%)

**Abbreviations:** UIP, Usual Interstitial Pneumonia; IPF, Idiopathic Pulmonary Fibrosis; CPFE, Combined Pulmonary Fibrosis and Emphysema; NSIP, Nonspecific Interstitial Pneumonia; HP, Hypersensitivity Pneumonia, ILD, Interstitial Lung Disease; FVC, Forced Vital Capacity; DLCO, Diffusion Lung Carbon Oxide; 6mWT, 6 min walking test.

**Table 2:** Average thickness of the pleural line in the different groups of patients.

Thickness of the Pleural Line (mm)				
Honeycombing (n = 32)		No Honeycombing (n = 11)		*p* Value
4.70 ± 0.65		4.62 ± 0.56		>0.05
Ground Glass (n = 5)	Honeycombing (n = 28)		Mixed (n = 10)	*p* Value
4.87 ± 0.59	4.75 ± 0.60		4.63 ± 0.71	>0.05
Minimal (n = 4)	Mild (n = 16)	Moderate (n = 10)	Severe (n = 13)	*p* Value
3.58 ± 0.18	4.19 ± 0.30	4.91 ± 0.32	5.45 ± 0.52	<0.0001

**Table 3:** Presence of >3 B-lines between different degrees of fibrosis.

>3 B-Lines				
Minimal (n = 4)	Mild (n = 16)	Moderate (n = 10)	Severe (n = 13)	*p* Value
3 (75%)	14 (88%)	8 (80%)	13 (100%)	>0.05

**Table 4:** Presence of TUS subpleural nodules between different HRCT patterns.

Subpleural Nodules			
Honeycombing (n = 28)	Reticulo-Nodular Pattern (with Honeycombing) (n = 4)	Reticular Pattern (without Honeycombing) (n = 5)	*p* Value
23 (82%)	3 (75%)	5 (100%)	>0.05

## Data Availability

The data presented in this study are available on request from the corresponding author. The data are not publicly available due to ethical reasons.
